# Soft ionization mechanisms in flexible µ-tube plasma—elucidation of He-, Ar-, Kr-, and Xe-FµTP

**DOI:** 10.1007/s00216-024-05419-1

**Published:** 2024-07-15

**Authors:** Caiyan Tian, Hao Song, Norman Ahlmann, Sebastian Brandt, Daniel Foest, Guanghui Niu, Joachim Franzke, Luisa Speicher

**Affiliations:** 1 Leibniz-Institute for Analytical Sciences – ISAS – eV., Bunsen-Kirchhoff-Straße 11, 44139 Dortmund, Germany; 2 Laboratory of Inorganic Chemistry, Department of Chemistry and Applied Biosciences, ETH Zurich, Vladimir-Prelog-Weg 1, 8093 Zurich, Switzerland

**Keywords:** Flexible µ-tube plasma (FµTP), Noble gas ions, Mechanisms of soft ionization

## Abstract

The soft ionization mechanism of helium-based plasma seems to be understood while it still remains challenging in argon-based plasma, although many studies have used argon plasmas as a soft ionization source with good ionization efficiencies. In this study, helium, argon, krypton, and xenon were fed into the same discharge geometry, a flexible micro-tube plasma (FµTP), to determine the ionization mechanisms. The FµTPs operated with the named noble gases obtained comparable ionization efficiencies by MS measurements. The optical emission results showed that N_2_^+^ were the dominant ions within the helium-FµTP and noble gas ions were dominant for the other plasmas. These ions support the development of excitation and eventually stop at the end of the capillary. Therefore, Penning ionization and charge transfer between plasma and ambient air/analytes in the open atmosphere have been proven not to be the primary soft ionization mechanism. Furthermore, it was found that photoionization played a minor role in soft ionization. Using helium as a diagnosis gas in front of the discharge capillary nozzle of the FµTP, where the sample is usually positioned, shows that helium can be ignited by all of these FµTPs. This demonstrates that the excitation of a diagnosis gas as well as the ionization of analytes is independent of the type of the discharge gas. An alternative mechanism that a transient potential created by the ions is responsible for the soft ionization is subsequently proposed.

**Supplementary Information:**

The online version contains supplementary material available at 10.1007/s00216-024-05419-1.

## Introduction

The first studies on soft ionization of molecules using a corona discharge in air at atmospheric pressures were reported by Shahin in 1966 [1]. He hypothesized that the primary ions of a discharge (e.g., N_2_^+^, O_2_^+^) might be responsible for the formation of (H_2_O)_n_ H_3_O^+^ ions by the production of either a hydride or through a charge exchange process. In the case of the production of a hydride, such as N_2_H^+^ or O_2_H^+^, a reaction would proceed as follows:

1 $${\text{N}}_{2}^{+}+ {\text{H}}_{2}\text{O }\to {\text{N}}_{2}{\text{H}}^{+}+\text{OH}$$

This is followed by the collision of the hydride with water to obtain an H_3_O^+^

2 $${\text{N}}_{2}{\text{H}}^{+}+{\text{H}}_{2}\text{O }\to {\text{H}}_{3}{\text{O}}^{+}+\text{N}_{2}$$

In the case of the charge exchange process, the reaction is

3 $${\text{N}}_{2}^{+}+ {\text{H}}_{2}\text{O }\to {\text{N}}_{2}+ {\text{H}}_{2}{\text{O}}^{+}$$

and the subsequent reaction

4 $${\text{H}}_{2}{\text{O}}^{+}+{\text{H}}_{2}\text{O }\to {\text{H}}_{3}{\text{O}}^{+}+\text{OH}$$

In both cases, the first member of the series (H_2_O)_n_ H_3_O^+^ is obtained, while the former case is a more likely pathway [1].

Four years later in 1970, Good et al. [2] described ion molecule reactions in pure nitrogen and nitrogen containing traces of water at a total pressure of 0.5 to 4 torr and the kinetics of clustering reactions forming (H_2_O)_n_ H^+^, in which the following reactions were involved:

3a $${\text{N}}_{2}^{+}+ {2\text{N}}_{2} \to {\text{N}}_{4}^{+}+{\text{N}}_{2}$$

3b $${\text{N}}_{4}^{+}+ {\text{H}}_{2}\text{O }\to {2\text{N}}_{2}+{\text{H}}_{2}{\text{O}}^{+}$$

4 $${\text{H}}_{2}{\text{O}}^{+}+{\text{H}}_{2}\text{O }\to {\text{H}}_{3}{\text{O}}^{+}+\text{OH}$$

5 $${\text{H}}_{3}{\text{O}}^{+}+{\text{H}}_{2}\text{O}+ {\text{N}}_{2} \to {\text{H}}_{3}{\text{O}}^{+}{{(\text{H}}_{2}\text{O})}_{1}+{\text{N}}_{2}$$

6 $${\text{H}}_{3}{\text{O}}^{+}{{(\text{H}}_{2}\text{O})}_{1}+{\text{H}}_{2}\text{O}+ {\text{N}}_{2} \to {\text{H}}_{3}{\text{O}}^{+}{{(\text{H}}_{2}\text{O})}_{2}+{\text{N}}_{2}$$

It was argued that the production of H_3_O^+^ happens almost exclusively through reactions (3b) and (4) [2].

In 1973, Horning et al. [3] published a detection system based on a mass spectrometer with an external ionization source containing a Ni.^63^ source at atmospheric pressure using nitrogen as the carrier gas. They cited the above given equations applicable for pressures between 0.5 and 4 torr of Good et al. and added the very first equation here called (0)

0 $${\text{N}}_{2}+{\text{e}}^{-} \to {\text{N}}_{2}^{+}+2{\text{e}}^{-}$$

Since then, the equations were cited in mass spectrometry or ion mobility textbooks or publications, whenever soft ionization or protonation is explained, even if experiments were carried out in ambient air.

In 2007, the first spectroscopic characterization of a dielectric barrier discharge was published, where He was fed as the buffer gas and a plasma jet was visible outside the capillary [4]. Spatially resolved spectroscopic emission measurements showed very intense N_2_^+^ lines in the plasma jet. Due to the knowledge of [1–3], it was assumed that these N_2_^+^ ions are mainly responsible for the protonation of molecules and sustain the production of primary ions. This source was developed to be an ionization source for gaseous compounds under atmospheric pressure as an alternative to traditional APCI or Ni^63^ β emitting source [4].

A low-temperature plasma (LTP) probe [5] based on dielectric barrier discharge was characterized by means of optical spectroscopy [6]. A completely different gas-phase chemistry was observed when the plasma gas was changed from He to Ar. The lack of N_2_ and N_2_^+^ emission in Ar-LTP argues that the excited-state chemistry of He is crucial for the formation of reactive nitrogen species for ambient mass spectrometry, which further correlates with mass spectral data acquired with an Ar-LTP probe that often yields 10 to 100 times weaker ion signals for an analyte of interest. This could not totally be stated by spatially resolved spectroscopic measurements of a dielectric barrier discharge plasma jet [7]. In case of an Ar dielectric barrier discharge, the N_2_^+^ ions were not measurable, but the emission of the excited N_2_ 357 nm could because the upper states could be excited by collisions of N_2_ with Ar metastables (Ar^M^) [7].

Although a variety of discharge gases have been used with sources like DART [8], FAPA [9], and LTP [5], higher sensitivities for a wider range of analyte ions were achieved using He as a discharge gas, rather than Ar, nitrogen, or air [10]. This finding is often attributed to long-lived, high-energy He metastables (He^M^), which can ionize most analytes (M) directly by Penning ionization [8]:

7 $${\text{He}}^{\text{M}}+\text{M }\to \text{He}+{\text{M}}^{+}+\text{e}^{-}$$

It has also been proposed [9] that He^M^ could ionize atmospheric gases, such as nitrogen or water vapor to produce reagent ions:

8 $${\text{He}}^{\text{M}} +{\text{N}}_{2} \to \text{He}+{\text{N}}_{2}^{+}+{\text{e}}^{-}$$

9 $${\text{He}}^{\text{M}} +{\text{H}}_{2}\text{O }\to \text{He}+{{\text{H}}_{2}\text{O}}^{+}+{\text{e}}^{-}$$

In Ar plasma, the direct Penning ionization between Ar^M^ with long lifetime and analyte can happen like reaction (7) if the energy difference of them is low enough, but the energies of Ar^M^ (11.5 and 11.7 eV) are insufficient to ionize N_2_ (15.6 eV) and H_2_O (12.6 eV) molecules and therefore to produce the H_2_O^+^ by Penning ionization. The ionization mechanism for the generation of protonated analytes using Ar plasma is not fully understood.

However, more and more plasmas developed for soft ionization have been operated with Ar instead of He in recent years. It has been shown that the ionization efficiency, i.e., the limit of detection (LOD), with Ar plasmas is similar to that with He-driven plasmas, in some cases even better [11, 12]. Different hypotheses have been proposed to explain the protonation of analytes.

Bogaerts [13] suggested that Ar^+^ and Ar_2_^+^ ions may be generated in Ar plasmas. These ions might have enough energy to ionize H_2_O molecules forming H_2_O^+^ or react with the analyte by charge transfer. In addition, the VUV photons radiated from any of Ar excited states, which locate at higher level than the ionization level of H_2_O molecules or analyte, may also ionize the H_2_O molecules or analyte [11].

In this work, not only He and Ar but also Kr and Xe were used as discharge gas to a flexible µ-tube plasma (FµTP) ionization source, which allows to sustain plasmas with different noble gases without changing the geometry with only small changes in the applied voltages. On the one hand, the ionization efficiencies of these plasmas for several analytes were evaluated by coupling to a mass spectrometer. At the same time, it was compared with the commercial photoionization lamps replacing FµTPs as the ionization source. On the other hand, optical characterization was performed to identify the reactive species involved in these plasmas and to investigate their propagation mechanism along the discharge capillary, which provides further insight into the soft ionization of plasma-based ionization sources.

## Material and methods

### Chemicals and solvents

The analytes used were mesitylene (Mes) obtained from Sigma-Aldrich with an ionization energy (IE) of 8.4 eV, acetone (Ace) from Honeywell with 9.7 eV, 2-propanol (IPA) from Roth with 10.2 eV, and acetonitrile (AceN) from Roth with 12.2 eV being close to the water ionization energy (12.6 eV). Nitrogen purchased from Westfalen Austria GmbH, Germany, was used as the reactant gas.

### FµTP and photoionization lamps

The FµTP source consists of a high voltage tungsten wire electrode inserted into a fused silica capillary tube without using any counter electrode as previously described [14]. The distance between the electrode tip and the capillary end was 3 mm in this work. The device was driven by a home-built square wave generator providing a maximum voltage of 3.5 kV. A frequency of 20 kHz and a duty cycle of 50:50 were fixed. He, Ar, and Kr with the purity of 99.999% and Xe with the purity of 99.99% purchased from Westfalen Austria GmbH, Germany, were used to evaluate the ionization mechanism. The gas flow was controlled by a mass flow controller (ALICAT).

DC photoionization lamps driven by Kr (type PKS106 by Heraeus) and Xe (type PXS096 by Heraeus) and RF photoionization lamps driven by Kr and Xe were used. The RF-Kr lamps have 13 MHz (type PKR106 by Heraeus) and 100 kHz (type PKR106-6 by Heraeus) and the RF-Xe lamp 13 MHz (type PXR096 by Heraeus). Those DC lamps were run by a home-built generator working from 0.1 to 2 mA and the RF lamps were run by commercial generators type C220S by Heraeus for the 100-kHz lamp and type C210 by Heraeus for the 13-MHz lamps. These lamps have magnesium fluoride windows to realize a sufficient transmission from the inside of the lamp to ambient air.

### Mass spectrometer

**Fig. 1:**
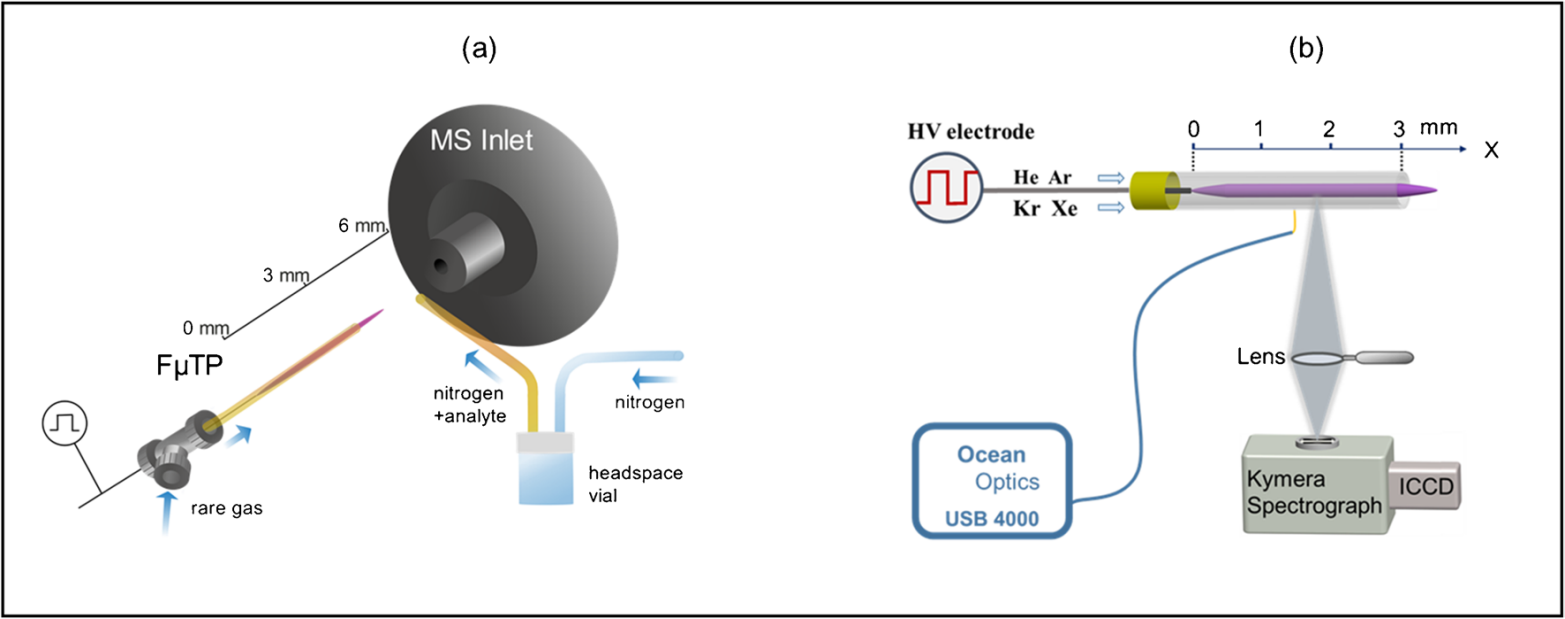
Experimental arrangement **a** for mass spectrometric measurements and **b** for optical emission measurements

A Finnigan LTQ mass spectrometer by Thermo Fisher was used to measure the ionization efficiency of the respective plasma sources. The capillary was heated to 200 °C, while the capillary voltage was set to 11 V and the tube lens to 35 V. Those parameters were chosen after a tune on the (H_2_O)_2_H^+^ peak with a mass of m/z 37 was done. Between the MS inlet and the plasma placed 3 mm away, a fused silica capillary was adjusted perpendicular as an outlet for the head space analytes supply with 100 mL min^−1^ N_2_ as a reactant gas. The reactant gas was applied to a vial with the liquid sample in it. The balanced vapor pressure is used as the headspace gas. A FµTP was used as an ionization source coupled to a mass spectrometer as shown in Fig. 1a. The plasma was replaced by the named lamps for the investigation of photoionization.

### Optical emission spectrometers

A USB 4000 spectrometer (USB 4000, Ocean Optics) with an optical fiber was used to obtain the reactive species present in these plasmas. The resolution of this spectrometer is 0.15 nm. The spectral range is 300–900 nm.

A fast intensified charge coupled device (ICCD camera, Andor DH 720 18F-03) captures the temporally and spatially resolved spectra from a Kymera adaptive focus imaging spectrograph (Kymera 193i). A diffraction grating of 1200 grooves mm^−1^ was chosen. The plasma emission was imaged on an ICCD camera through a convex lens with a focal length of 75 mm. The arrangement for optical measurements is shown in Fig. 1b.

For all discharge gases, a gas flow of 75 mL min^−1^ and a voltage of 3.0 kV were used for the mass measurements. The detailed experimental parameters for optical measurements are listed in Table S1.

### Data acquisition and processing

The data measured for each spectrum by the MS are an average of 1 min acquiring in full scan mode. The generated spectra were extracted as raw files from Xcalibur Qual browser to a txt file. Subsequently, the data was visualized using Python.

Various wavelengths were measured by the ICCD camera. The selection of these wavelengths was performed by changing the central wavelength of the spectrometer with the help of Andor Solis software. The obtained data using Andor Solis software are in the SIF file format. The detailed processing procedure from raw data to the contour form can be found in our previous work [15].

## Results and discussion

### Mass spectrometry

The ionization efficiency of FµTPs using different noble gases as plasma gases and the efficiency of commercial photoionization lamps were examined by coupling with a mass spectrometer. The results are presented and discussed in the following.

### Ionization by FµTP applied with different noble gases as plasma gas

**Fig. 2:**
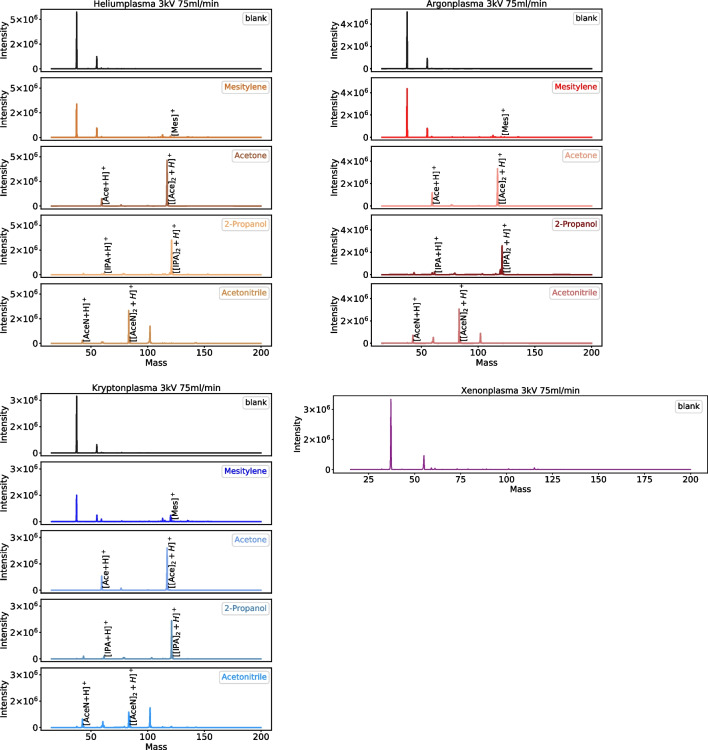
Blank spectra and analyte spectra of mesitylene, acetone, 2-propanol, and acetonitrile when the ionization sources He-, Ar-, and Kr-FµTP are used. Blank spectrum when a Xe-FµTP is applied

Figure 2 shows mass spectra recorded with the He-, Ar-, and Kr-FµTP as ionization source showing the m/z ratio on the abscissa and the intensity on the ordinate. The spectra are normalized to the respective highest intensities. The respective upper graphs show spectra where no analyte was used with the water peaks [H_2_O]_2_ H^+^ at m/z 37 and [H_2_O]_3_ H^+^ at m/z 55. Then, from top to bottom, the mass spectra for mesitylene, acetone, 2-propanol, and acetonitrile are as follows. The mesitylene spectra obtained with each FµTP show the water peaks mentioned above, but only a very small mesitylene [Mes]^+^ peak at m/z 120. Each acetone and 2-propanol spectrum shows two peaks, [Ace + H]^+^ at m/z 59 and [[Ace]_2_ + H]^+^ at m/z 117, [IPA + H]^+^ at m/z 61 and the second one [[IPA]_2_ + H]^+^ at m/z 121, respectively. Four peaks are visible in the acetonitrile spectra, one is [AceN + H]^+^ at m/z 42, the others are [AceN + H_2_O + H]^+^ at m/z 60, [[AceN]_2_ + H]^+^ at m/z 83, and [[AceN + H_2_O]_2_ + H]^+^ at m/z 101. In addition, a blank spectrum is shown in Fig. 2 when a Xe-FµTP was deployed and shows the water peaks [H_2_O]_2_ H^+^ (m/z 37) and [H_2_O]_3_ H^+^ (m/z 55) with an intensity of 3.5 × 10^6^ and 7.0 × 10^5^. As Xe is a very expensive gas, the Xe-FµTP source was not used for the measurements of the ionization efficiency of analytes.

**Fig. 3:**
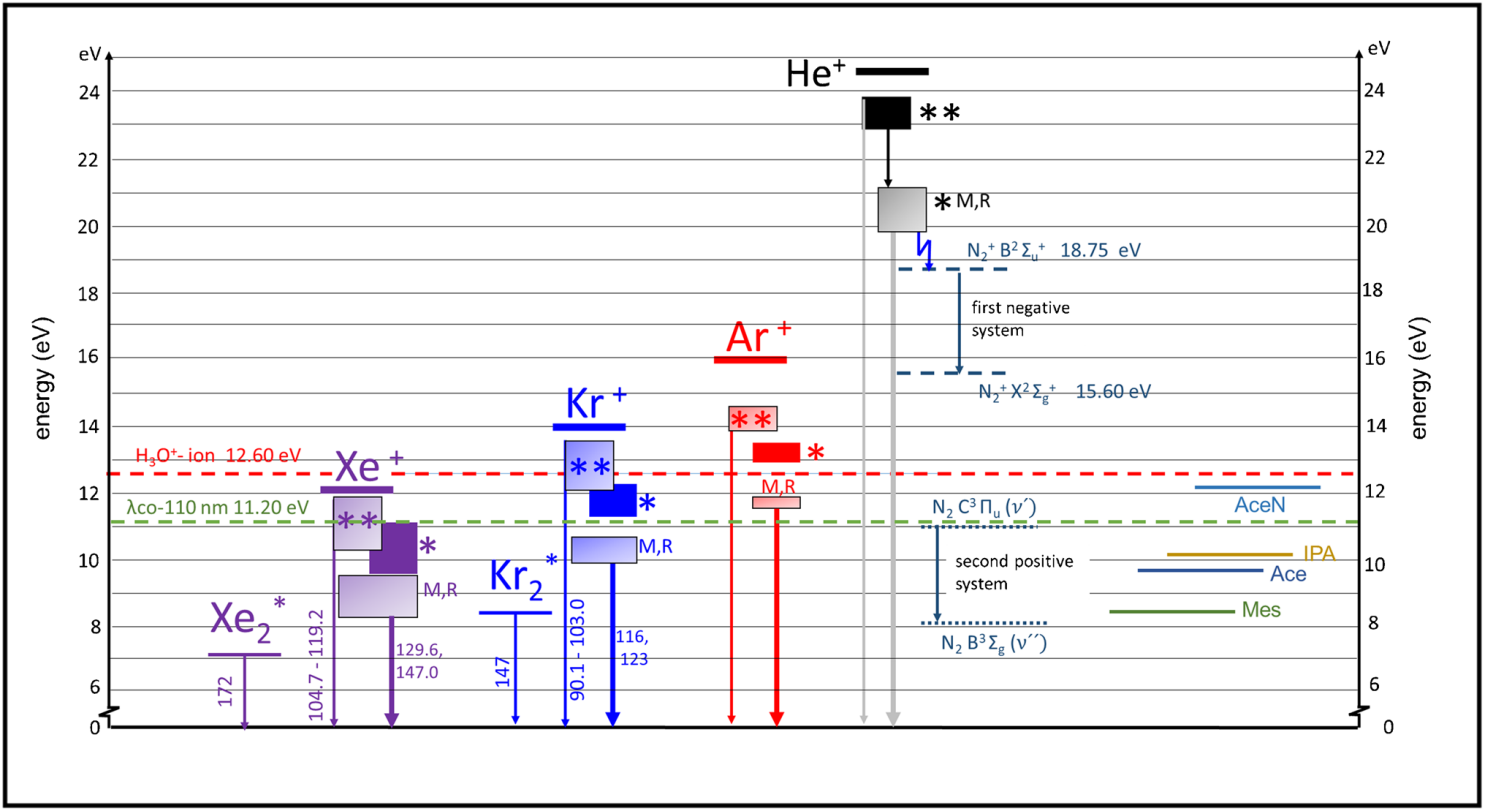
Diagram of the excitation and ionization energy for different noble gases and related nitrogen species, and ionization energy of analytes. Nitrogen first negative system (FNS) and nitrogen second positive system (SPS). λco-110 nm refers to the cut-off wavelength

The intensities of all mentioned peaks are shown in Table 1. Due to the delivery through headspace, where higher concentrations of the analyte may enter the ionization region, the intensities of all corresponding clusters such as dimers are higher than these of the protonated analyte. The fields with the highest and the second highest intensities for one analyte peak are marked with red and blue, respectively. The intensities are very similar for all FµTPs; it is therefore independent of the type of the noble gas the FµTP is operated with. This cannot be explained by the usual explanation of Penning ionization or charge transfer, which takes place between the pure noble gas plasmas and the ambient air/analyte. Since both mechanisms base on the fact that a species with higher energy collides with one in the ground state, which can be excited or ionized in a state lower than that of the colliding partner. In the case of He, the metastable state (He^M^) fulfils this condition laying higher than the N_2_^+^ B^2^ Σ_u_^+^ and the H_2_O dissociation level to produce H_3_O^+^ as shown in Fig. 3. In the case of Ar, only the ionization level Ar^+^ is higher than N_2_^+^ X^2^ Σ_g_^+^ and the H_2_O dissociation level, but the lifetime of an Ar^+^ is much shorter than that of metastables due to recombination and optical transitions to lower states. In the case of Kr, the Kr^+^ would be sufficiently high to ionize only H_2_O but not high enough to ionize N_2_. Last, but not least, the Xe^+^ level is not high enough to ionize N_2_ nor to dissociate any water which is clearly shown in Fig. 3. Therefore, other mechanisms must be responsible for the ionization of the analyte.

**Table 1:**
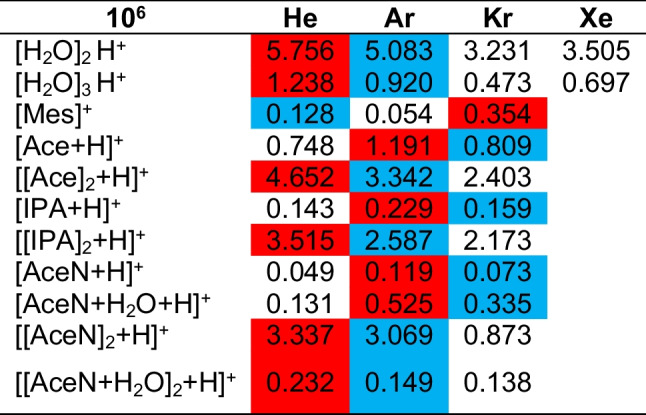
Intensities for the measured peaks for a blank signal and the analytes mesitylene, acetone, 2-propanol, and acetonitrile obtained with He-, Ar-, Kr-, and Xe-FµTP. Marked in red are the highest intensities for each analyte, in blue the second highest

### Contribution of photoionization

The most obvious explanation is that the mechanism, which is relevant for soft ionization by the FµTP could be due to photoionization by the radiation leaving the FµTP. To investigate the impact of photoionization, experimental arrangements with commercially available photoionization Kr and Xe lamps instead of the plasma sources were applied. A difference of the photoionization lamps and the FµTP is the applied pressure, in the case of the lamp the pressure is about 10 mbar, in the case of the FµTP 1 bar. At gas pressure higher than 300 mbar, the formation of excimer state is increasing [16, 17]. Therefore, the VUV transitions are atomic transitions for the lamp and more excimer transitions for the FµTP. The relevant transitions in particular for the Kr and Xe lamps are shown in Fig. 3 marked by one arrow starting from the resonant states (R) or excited states marked with two stars to the appropriate ground state. The respective wavelengths and energies radiated from the resonant states are Kr 116 nm (10.69 eV) and 123 nm (10.08 eV) and Xe 129 nm (9.61 eV) and 147 nm (8.43 eV), which are too low to ionize the water molecule (12.6 eV). The radiation from excited states marked by two stars to the appropriate ground state emits in the wavelength range of 90.1–103.0 nm (13.76–12.04 eV) in the case of Kr, where certain energies are sufficient to ionize water molecules producing H_2_O^+^, while in the case of Xe, it is still not high enough to produce H_2_O^+^. As these lamps are equipped with an MgF_2_ window, wavelengths below 110 nm (higher than 11.27 eV) are cut off. Therefore, neither the radiation of a Kr lamp nor a Xe lamp is able to photoionize water directly. The relevant transitions in the case of the FµTP are more excimer transitions with emission wavelength of 146 nm and 172 nm for Kr_2_* and Xe_2_*, respectively. No photoionization of water can certainly take place with radiation of these wavelengths.

**Fig. 4:**
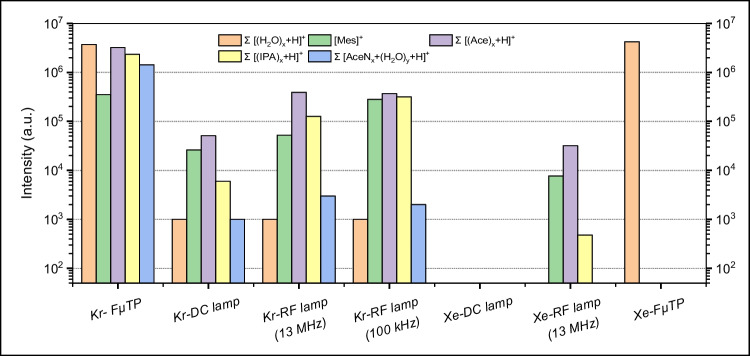
Comparison of mass intensities for the measured peaks for a blank signal and the analytes mesitylene, acetone, 2-propanol, and acetonitrile. Measured intensity in logarithmic (log) scale for the different ionization sources. Intensities under 10^2^ are stated as 0. The Xe-FµTP source was only used for the measurements of the black spectrum

The mass spectra using different lamps are shown in Figure S1 and S2 and the original intensities of all mentioned peaks are shown in Table S2. Their summarized intensities are compared with those obtained with the Kr- and Xe-FµTP sources. The results are shown in Fig. 4. It is striking that the mass intensities obtained by the ionization with the lamps is minimum one order of magnitude lower than these of the spectra with the FµTPs. That is, the maximum contribution of photoionization in FµTPs is 10%. Nevertheless, the soft ionization efficiency of the Kr- and Xe-FµTP plasmas as shown in Fig. 2 is comparable to those of the plasmas driven with the other noble gases. Therefore, the photoionization might play a minor role in the explanation for the soft ionization mechanism in the FµTP.

On the basis of the above analysis, there must be other mechanisms responsible for the soft ionization that differ from those of Penning ionization, charge transfer, and photoionization occurring between the pure noble gas plasmas and the analyte. In order to gain further insight into the soft ionization mechanism, it is beneficial for the individual noble gas-FµTP source to be investigated. In the following section, the optical characterization of these plasmas was carried out. The discharge gas flow of 50 mL min^−1^ was used.

### Emission spectrometry

The optical emission spectra in FµTP using different noble gases were obtained by a USB 4000 spectrometer to identify which reactive species are involved in individual plasma, while the temporally resolved emission of related species was investigated.

### Emission spectra of species in FµTP with different plasma gases

**Fig. 5:**
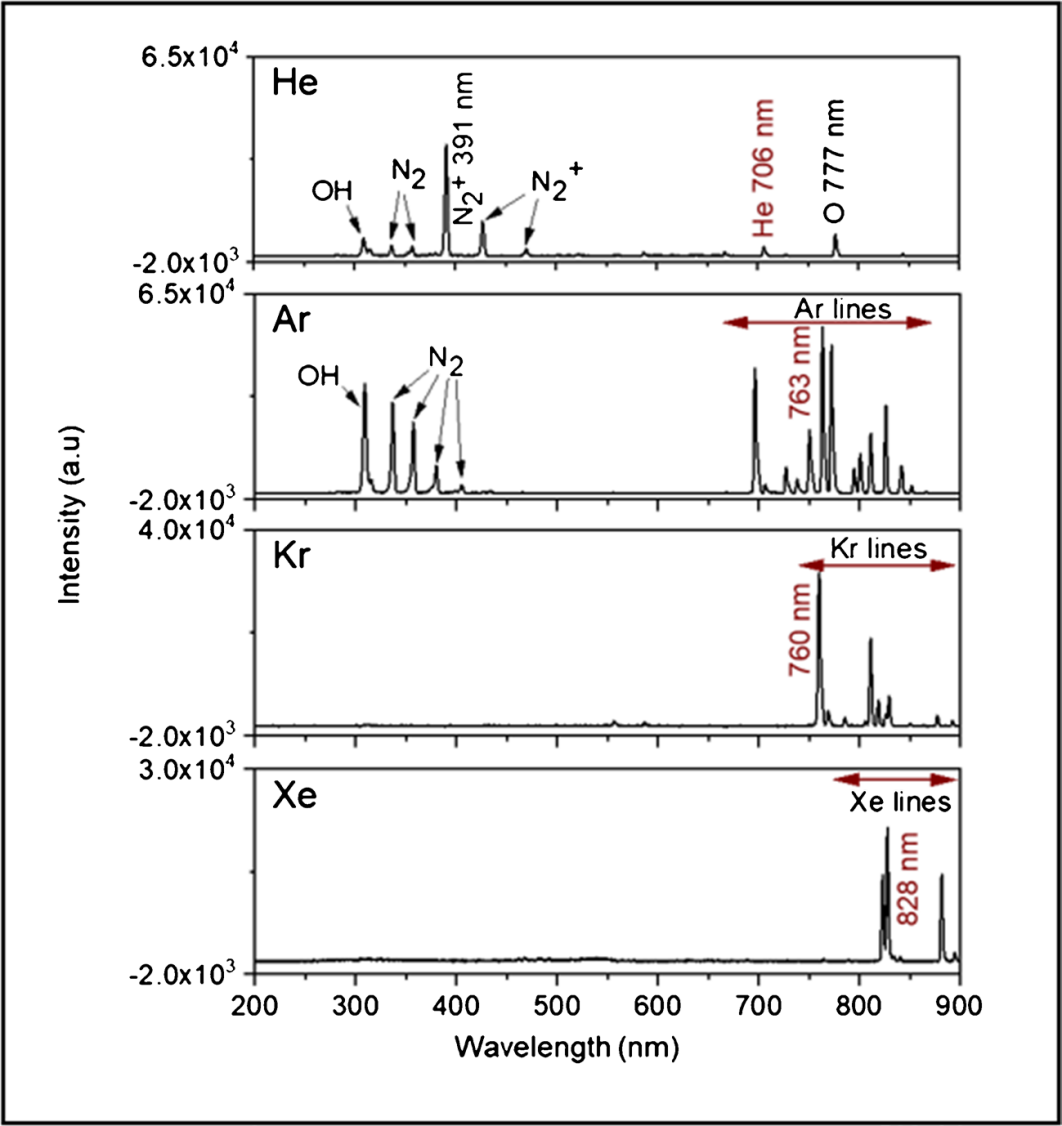
The emission spectra of He-, Ar-, Kr-, and Xe-FµTP in the plasma capillary at *x* = 1.5 mm. The applied voltages are 2.5, 3.5, 3.5, and 3.3 kV, respectively. The Xe plasma had a grounded ring around the capillary in the vicinity of the electrode

Figure 5 shows typical emission spectra of a He-, Ar-, Kr-, and Xe-FµTP measured in the range from 300 to 900 nm by an Ocean Optics spectrometer. The *X*-axis starts at 200 nm for a clear view of wavelengths around 300 nm. The optical fiber was placed in the middle of the plasma capillary at *x* = 1.5 mm.

The spectra of the transitions from the upper excited noble gas level N* to the lower-lying noble gas levels N^M/R^, or in the case of He from the upper excited He level He** to the lower excited He level He* which is coupled to the even lower He metastable (He^M^) level, are observed and marked in the spectra with red arrows, in which the wavelengths of He 706 nm, Ar 763 nm, Kr 760 nm, and Xe 828 nm are the transitions with the highest measured intensities of each noble gas. Except for Xe 828 nm, where a transition to a resonant state (N^R^) was chosen due to a higher intensity, transitions to a metastable state (N^M^) were used for the other noble gases.

The emission of the OH band, the second positive system (SPS) (N_2_ C ^2^Π_u_ → N_2_ B ^3^Π_g_) of N_2_ and the first negative system (FNS) (N_2_^+^ B ^2^Σ_u_^+^  → N_2_^+^ X ^2^Σ_g_^+^) are observed in He-FµTP. N_2_^+^ 391 nm is the dominant wavelength, since the He^M^ level is higher than that of the N_2_^+^ B ^2^Σ_u_^+^ and the energy difference between these states is 1.11 eV, which is beneficial for Penning ionization. In Ar-FµTP, the energy of Ar^M^ is much lower than that of the N_2_^+^ B ^2^Σ_u_^+^ state, as a result, N_2_^+^ B ^2^Σ_u_^+^ cannot be generated by Penning ionization, but the N_2_ C ^2^Π_u_ could be populated. For the Kr- and Xe-FµTP, the energies of both N^M^ are lower than those of N_2_ C ^2^Π_u_ and N_2_^+^ B ^2^Σ_u_^+^ states; therefore, neither SPS nor FNS is generated.

Since the soft ionization takes place outside of the capillary where usually a plasma jet is situated and there is only a very small plasma visible outside the capillary in case of a FµTP, a study of the generation and development mechanism of the plasma from the inside of capillary to the outside is valuable for soft ionization.

### Temporally resolved emission spectra for He-, Ar-, Kr-, and Xe-FµTP

**Fig. 6:**
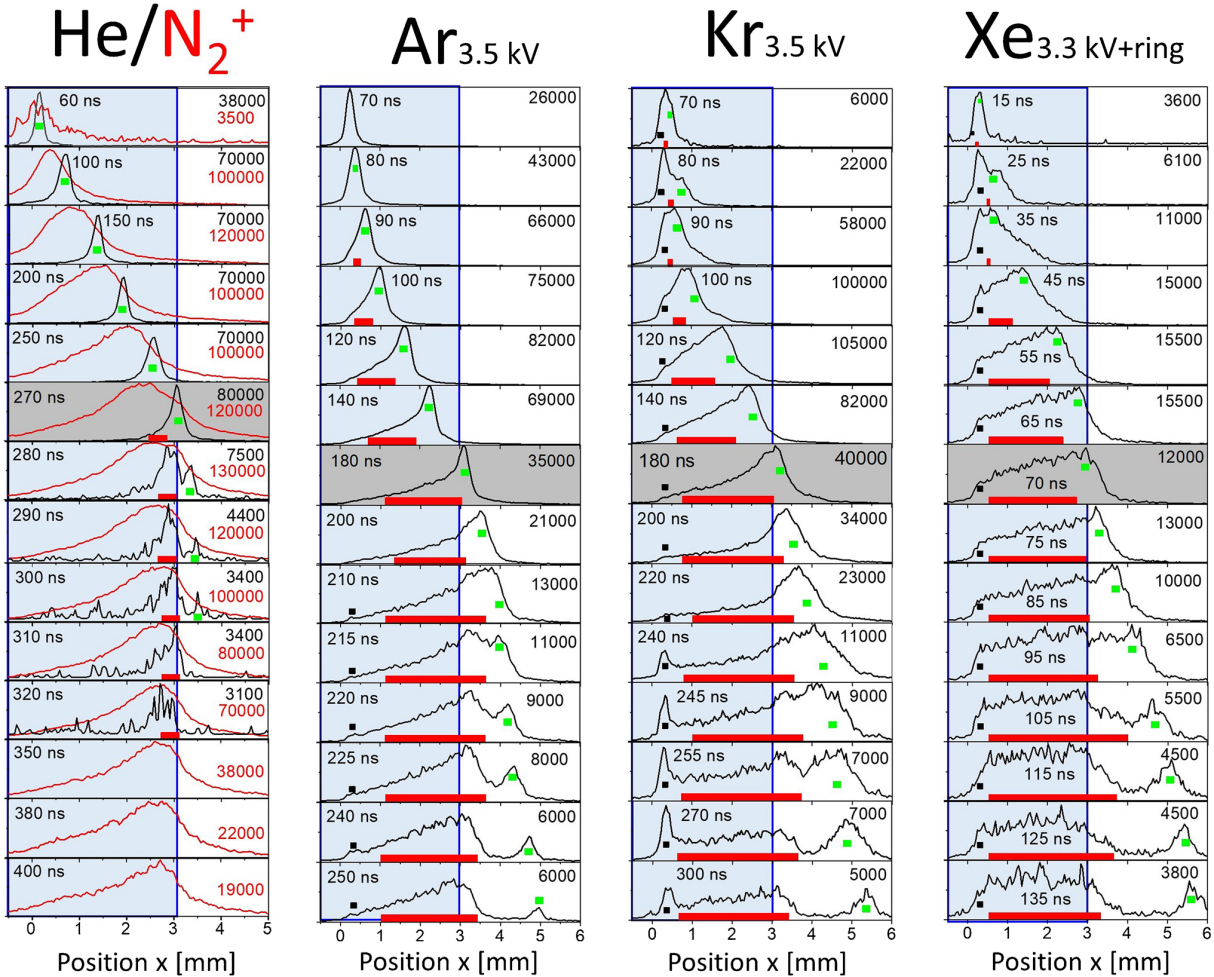
Plots for the emission intensities as a function of position at instants of time for the He-, Ar-, Kr-, and Xe-FµTP. He 706 nm and N_2_^+^ 391 nm, Ar 763 nm, Kr 760 nm, and Xe 828 nm

In Fig. 6, the emission intensities of He 706 nm (black line) and N_2_^+^ 391 nm (red line) in the left plot, as well as Ar 763 nm, Kr 760 nm, and Xe 828 nm in the corresponding subplots are presented as a function of position at different instants of time normalized to the maximum of each spectrum. The position *x* = 0 mm represents the electrode tip and the nozzle of the plasma capillary is at *x* = 3 mm, with further positions *x* > 3 mm being outside the capillary in the ambient atmosphere. The measured maximal intensities are inserted in the upper right corner and the instants of time are given in ns in each subplot.

In the case of He-FµTP, the He 706 nm and N_2_^+^ 391 nm spectra are given to show the propagation of excited He species as well as the N_2_^+^ species, which are relevant for the propagation of the plasma. The N_2_^+^ ion wave is following the He** excitation wave and the shapes of He 706 nm are nearly symmetric inside the capillary. A small wing on the left side is visible in the subplot at 270 ns (gray field), which is marked with a red bar. The wing might be attributed to the He^+^. In opposite to the excited and even higher excited states of He, the He^+^ is charged and therefore is to be expected at another position then the He**. In this sense, electron excited He can be distinguished from excited He which are in the process of recombination. The idea behind this explanation is that He^+^ could be depopulated by IR emission to the He**, and the He 706 nm signal corresponds to the transition from 3 s ^3^S_1_ (He**) to the 2P°_2,1,0_ (He*) states. Therefore, not only He** but also He^+^ can be measured, which locate at different positions of the He 706 nm signal. These states are generated by collision of electrons with He atoms, in which the electrons are accelerated in the electric field created by the applied voltage on the electrode tip in the beginning (*x* = 0 mm). The 2P°_2,1,0_ state is radiatively connected to the metastable state 2 s ^3^S_1_ (He^M^). These He^M^ collide with N_2_ X ^1^Σ_g_^+^ producing N_2_^+^ B ^2^Σ_u_^+^, which then decays to the N_2_^+^ X ^2^Σ_g_^+^ state resulting in the detectable signal of N_2_^+^ 391 nm. Therefore, the He 706 nm peak is always measured in front of the N_2_^+^ 391 nm peak (higher *x*) at the same moment.

With N_2_^+^, a new potential is formed in front of the electrode and is responsible for the next round of excitation of He and even ionization of He at a further position (*x* > 0 mm), where the He^M^ will collide with N_2_ molecules producing new N_2_^+^ ions, so that a further potential will be initiated. Such a circular process leads to a propagation of excited and ionized species through the capillary, which is in agreement with the report on the role of charge accumulation in plasma jets [18].

At 270 ns, the propagation of the excitation to the He** state reaches the end of the capillary. From 280 to 300 ns, the asymmetric signal of He 706 nm is separated into two signals, in which the one with the red bar stops at the end of capillary and the other with a green square propagates outside the capillary. Whereas the propagation of the N_2_^+^ 391 nm stops at the end of the capillary and its emission intensity starts decreasing from 280 ns, although with increasing *x* for *x* > 3 mm, the concentration of N_2_ is increasing and that of He is decreasing. Like the N_2_^+^, it is suspected that the wing is acted upon by He^+^, which also stop at the end of the capillary rather than move forward into ambient air. Nevertheless, the propagation of the He excitation to He** moves outside the capillary.

In the case of Ar-, Kr-, and Xe-FµTPs, N_2_^+^ B ^2^Σ_u_^+^ states will not be generated as explained above. Furthermore, the metastable and ionic levels of Kr and Xe are even lower than N_2_^+^ X ^2^Σ_g_^+^. Therefore, neither N_2_^+^ B ^2^Σ_u_^+^ nor N_2_^+^ X ^2^Σ_g_^+^ can be generated by collisions of these Kr or Xe species with the ground state of N_2_. Although the energy level of Ar^+^ is higher than the ionic energy of N_2_^+^ X ^2^Σ_g_^+^, the density of Ar^+^ is minimum one order of magnitude lower than the density of Ar^M^ and therefore the probability of ionization by charge transfer will be very low [19]. Therefore, in Ar-, Kr-, and Xe-FµTPs, neither N_2_^+^ B ^2^Σ_u_^+^ nor N_2_^+^ X ^2^Σ_g_^+^ play a role in promoting the excitation and ionization of plasmas inside the capillary.

Compared to the emission intensities as a function of position of He 706 nm, asymmetric shapes of the emission profiles of Ar 763 nm, Kr 760 nm, and Xe 828 nm are observed. These asymmetric shapes are due to a wing on the side to smaller *x* values marked by a red bar for nearly each instant of time, which resemble to those of the He 706 nm when the contribution of He^+^ to He 706 nm increases.

As He^+^ can be depopulated to He**, Ar^+^, Kr^+^, and Xe^+^ can also be depopulated by IR emission to the Ar*, Kr*, and Xe* states, respectively, and are detected by the same wavelength as the corresponding noble gas excited states. Therefore, the emission shapes of Ar 763 nm, Kr 760 nm, and Xe 828 nm show the contribution of their excited states (*) and ions to respective signals. Similar to He 706 nm, the wing is attributed to noble gas ions and the peak to excited neutrals, they are generated by collisions between the high-energy electrons and ground state atoms.

After 180 ns, 180 ns, and 70 ns, the propagation of the Ar, Kr, and Xe emissions, respectively, reaches the capillary outlet as shown in the subplots with the gray background. The excitation (green squares) for these three noble gas atoms propagates forward into the open air as shown at later instants of time while the ionization propagation (red bar) stops when the capillary end is reached and the signal decreases over time. Here, in the case of Ar-, Kr-, and Xe-FµTP, the separation of the ion signal (red bar) and the signal of the excited noble gases (green square) is more obvious than in the case of the He-FµTP.

Besides, there are obvious peaks marked by black squares in the vicinity of the electrode in Kr- and Xe-FµTP during the whole discharge process, and these peaks almost do not propagate. In Ar-FµTP, it is not that obvious in the first 200 ns and becomes clear at later times from 215 to 250 ns. These peaks are narrower than the other peaks measured, so that they cannot be assigned as ion peaks, but should be excited peaks.

### Proposed mechanism of excitation and ionization outside the capillary

A conclusion of the propagation processes inside the capillary is that the propagation of the excitation is in front of the propagation of the ionization. That means that the propagation of the ionization or in other words the propagation of ion production leads to an excitation of the noble gas atoms in front of the ion propagation front. Then the question arises as to why the excited noble gas atoms continue to move outside the capillary (*x* > 3 mm) even though the ionic propagation stops at the end of the capillary. When no charges would leave the capillary and therewith the potential would decrease in time, it could be expected that the excited noble gas atoms should decrease in time and remain at the same position or even move to lower *x*. On the contrary, they move to higher *x*. As described above, the further propagation of the excitation of noble gas atoms can only happen when the propagation of the ionization takes place. Since the propagation of the noble gas ions stops at the end of the capillary, other components must be ionized to continue the propagation of the excitation of the noble gas atoms outside the capillary. Since from the end of the capillary to higher *x* the concentration of the noble gases decreases and the components in ambient air increase, the species of the ambient air might be ionized, e.g., N_2_^+^ X ^2^Σ_g_^+^, H_3_O^+^ or O_2_^+^.

Unfortunately, these species cannot be measured by emission because no transitions of species of the ambient air are known or in the wavelength range of the used spectrometer. Once we tried to measure the density of the N_2_^+^ X ^2^Σ_g_^+^ state by diode laser absorption, it was not possible to measure any signal. This may be quenched by O_2_ and H_2_O in air. Therefore, a diagnosis gas He with flow rate of 500 mL min^−1^ was introduced via a diagnosis capillary without any electrode.

**Fig. 7:**
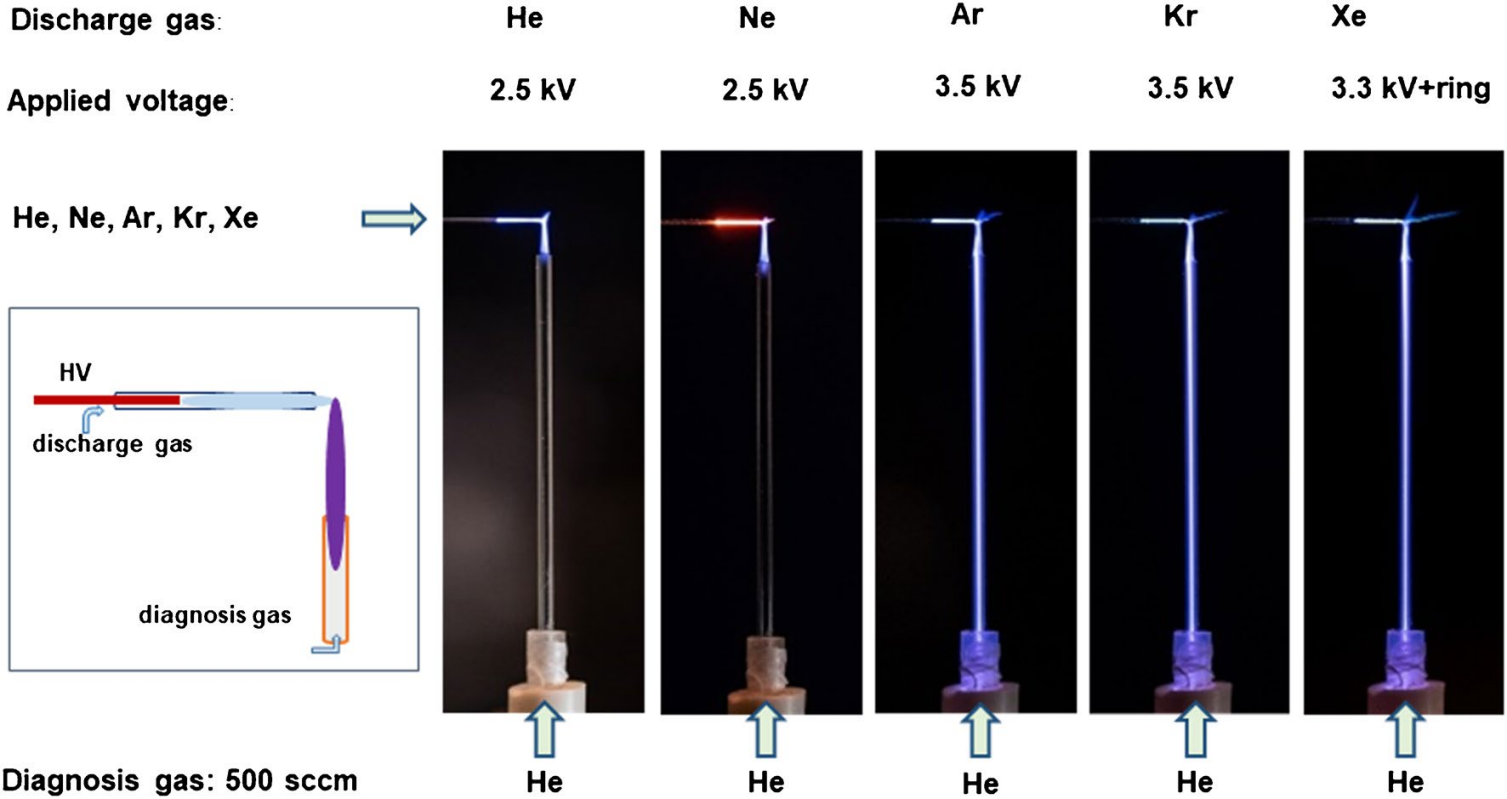
Photos of He-, Ne-, Ar-, Kr-, and Xe-FµTP with He as diagnosis gas in front of the outlet of the capillary

Figure 7 presents the propagation of the emission against the direction of the diagnosis gas flow when a FµTP is operated with noble gases such as He, Ne, Ar, Kr, or Xe and the diagnosis gas is He. Here, Ne is also used as a discharge gas to distinguish plasmas generated along the discharge capillary and diagnosis capillary clearly by the naked eye. The plasma along the discharge capillary is orange and the color of the emission along the diagnosis capillary has the color of a He plasma. It is comparable with that of the plasma obtained in the case of He–He plasma, where both discharge gas and diagnosis gas are He. This result demonstrates that the diagnosis gas He is excited by the Ne-FµTP. In addition, the diagnosis gas He is also excited by the Ar-, Kr-, and Xe-FµTP, where the emission spreads into the diagnosis tube. It is worth noting that all excited states and even the ionic states of the Ne-, Ar-, Kr-, and Xe-FµTPs are below the upper excited state of He (He**), which is responsible for the transition of He 706 nm signal. In this sense, the excitation of the diagnosis gas He is not dependent on the type of discharge gas, and therefore it is not attributable to Penning ionization or charge transfer.

Based on these findings, a reasonable interpretation is proposed that the ions arriving at the end of the capillary create a transient potential and therewith an electric field, in which He atoms of the diagnosis gas are excited to the He** state or even higher states. The height of this transient signal is not dependent on the type of discharge gas but on the number of charges. This is the only way to explain, why Ar, Kr, or even Xe with much lower excited or ionized states than He have are able to excite or even ionize He. If the diagnostic gas is replaced by ambient air, it can be understood that N_2_^+^ X ^2^Σ_g_^+^, H_3_O^+^, or O_2_^+^ can be ionized even if their ionization levels are higher than that of the noble gas used for the discharge. So, there are two possibilities to receive ions in the ambient air. One is that electrons will be accelerated in the electrical field towards the transient potential. On its way, ions will be produced by collisions. The other is that electrons are torn out of molecules or atoms which are in the electric field, producing ions and additional electrons.

## Conclusion

In this study, not only He and Ar but also Kr and Xe were fed as discharge gases to the same ionization source to study the soft ionization mechanism. The results with different plasmas demonstrated that a complex mechanism contributes to the soft ionization.

Optical emission spectroscopy measurements showed that N_2_^+^ is the dominant positive ion species formed in He-FµTP, whereas in Ar-, Kr-, and Xe-FµTP, N_2_^+^ is not observed but noble gas ions are identified. Most importantly, these ions stop at the end of the discharge capillary, and some of these noble gas ions have too low energy levels to form N_2_^+^ or even H_2_O^+^ by charge transfer. In the case of the He plasma, the ionization of analytes is usually explained by the generation of protonated water clusters produced through a series of reactions involving N_2_^+^, while it is not suitable for Ar, Kr, and Xe plasmas. Nevertheless, the mass spectrometry results with Ar-, Kr-, and Xe-FµTP showed comparable ionization efficiencies as those with He-FµTP. This reveals that Penning ionization or charge transfer between the plasma gases and ambient air/analytes is not the dominant ionization pathway for the generation of reagent ions. At the same time, the impact of photoionization on the soft ionization was evaluated by means of commercial lamps, which is determined that the maximum contribution of photoionization is 10%.

However, a diagnosis gas He can be ignited by an Ar-, Kr-, and Xe-FµTP even if all excited and even ionic states of these plasmas are lower than the excited ones of He. One possible interpretation is that a transient potential is created by the noble gas ions at the end of the discharge capillary and therewith an electric field. As a result, the excitation of the diagnosis gas and the generation of reagent ions in the open atmosphere are not dependent on the type of discharge gas. Such transient potential makes a significant contribution to the soft ionization. It is reasonable to expect that the diagnosis gas can still be ignited and the analyte can be ionized when a glass wall is present between the two bundles of gas. In this case, photoionization could be ruled out completely and the whole mechanism would work with the named potential. This assumption needs further experimental validation that will be given in the following work [Soft Ionization Mechanisms in Flexible µ-Tube Plasma—From FµTP to Closed µ-Tube Plasma] [20].

Although only one discharge configuration was used as a soft ionization source in the present study, these findings may potentially be applied to other plasma-based ionization sources and definitely should be considered in future studies.

## Supplementary Information

Below is the link to the electronic supplementary material.

Supplementary file1 (DOCX 451 KB)
