# Correction to: Soft ionization mechanisms in flexible μ‑tube plasma—elucidation of He‑, Ar‑, Kr‑, and Xe‑FμTP

**DOI:** 10.1007/s00216-024-05607-z

**Published:** 2024-10-18

**Authors:** Caiyan Tian, Hao Song, Norman Ahlmann, Sebastian Brandt, Daniel Foest, Guanghui Niu, Joachim Franzke, Luisa Speicher

**Affiliations:** 1 Leibniz-Institute for Analytical Sciences – ISAS – eV., Bunsen‑Kirchhoff‑Straße 11, 44139 Dortmund, Germany; 2 Laboratory of Inorganic Chemistry, Department of Chemistry and Applied Biosciences, ETH Zurich, Vladimir‑Prelog‑Weg 1, 8093 Zurich, Switzerland

**Correction to: Analytical and Bioanalytical Chemistry (2024) 416:4907–4918**

10.1007/s00216-024-05419-1

The article Soft ionization mechanisms in flexible μ‑tube plasma—elucidation of He‑, Ar‑, Kr‑, and Xe‑FμTP, written by Caiyan Tian, Hao Song, Norman Ahlmann, Sebastian Brandt, Daniel Foest, Guanghui Niu, Joachim Franzke, and Luisa Speicher, was originally published Online First without Open Access. After publication in volume 416, issue 22, pages 4907–4918 the author decided to opt for Open Choice and to make the article an Open Access publication. Therefore, the copyright of the article has been changed to © The Author(s) 2024 and the article is forthwith distributed under the terms of the Creative Commons Attribution 4.0 International License, which permits use, sharing, adaptation, distribution and reproduction in any medium or format, as long as you give appropriate credit to the original author(s) and the source, provide a link to the Creative Commons licence, and indicate if changes were made. The images or other third party material in this article are included in the article’s Creative Commons licence, unless indicated otherwise in a credit line to the material. If material is not included in the article’s Creative Commons licence and your intended use is not permitted by statutory regulation or exceeds the permitted use, you will need to obtain permission directly from the copyright holder. To view a copy of this licence, visit http://creativecommons.org/licenses/by/4.0/.

The original article has been corrected.

