# Supplementary material for: Soft ionization mechanisms in flexible µ-tube plasma—elucidation of He-, Ar-, Kr-, and Xe-FµTP
Source: Anal Bioanal Chem. 2024 Jul 15;416(22):4907–18. doi: 10.1007/s00216-024-05419-1 (PMC11480168; doi:10.1007/s00216-024-05419-1)
Supplement: Supplementary file 1 — Supplementary file1 (DOCX 451 KB) [file 216_2024_5419_MOESM1_ESM.docx]

**Supplementary Material to**

**Soft ionization mechanisms in** **flexible µ-tube plasma—elucidation of He-, Ar-, Kr-, and Xe-FµTP**

Caiyan Tian^a^, Hao Song^a^, Norman Ahlmann^a^, Sebastian Brandt^a^, Daniel Foest^a^, Guanghui Niu^b^, Joachim Franzke^a^*, Luisa Speicher^a^*

^a^Leibniz-Institute for Analytical Sciences – ISAS – eV., Bunsen-Kirchhoff-Straße 11, 44139 Dortmund, Germany

^b^Laboratory of Inorganic Chemistry, Department of Chemistry and Applied Biosciences, ETH Zurich, Vladimir-Prelog-Weg 1, 8093 Zurich, Switzerland

***** Corresponding author: Email: luisa.speicher@isas.de, and[joachim.franzke@isas.de](mailto:joachim.franzke@isas.de), Phone: +49 (0)2 31.13 92-293, and +49 (0)2 31.13 92-174

**Figure/table captions**

**Table S1** Experimental conditions for different plasma types

**Figure S1** Spectra of different DC photoionization lamps (Kr, Xe) used for the analytes Mesitylene, Acetone, 2-Propanol and Acetonitrile

**Figure S2.** Spectra of different RF ionization lamps (Kr, Xe) with the frequencies of 13 MHz and 100 kHz used for the analytes Mesitylene, Acetone, 2-Propanol and Acetonitrile

**Table S2**. Original intensities for the measured peaks for a blank signal and the analytes Mesitylene by DC and RF photo ionization lamps

**Table S1** Experimental conditions for different plasma types

| Plasma type | Applied voltage  (kV) | Gate width  (ns) | Step width  (ns) | Gain | Integration time  (ms) |
| --- | --- | --- | --- | --- | --- |
| He-FµTP | 2.5 | 10 | 10 | 250 | 30 |
| Ar-FµTP | 3.5 | 2.5 | 2.5 | 200 | 30 |
| Kr-FµTP | 3.5 | 2.5 | 2.5 | 250 | 30 |
| Xe-FµTP | 3.3+ring | 2.5 | 2.5 | 255 | 200 |

**Figure S1**. Spectra of different DC photoionization lamps (Kr, Xe) used for the analytes Mesitylene, Acetone, 2-Propanol and Acetonitrile.

**Figure S2.** Spectra of different RF ionization lamps (Kr, Xe) with the frequencies of 13MHz and 100kHz used for the analytes Mesitylene, Acetone, 2-Propanol and Acetonitrile.

**Table S2**. Intensities for the measured peaks for a blank signal and the analytes Mesitylene, Acetone, 2-Propanol and Acetonitrile for DC and RF photo ionization lamps Intensities under 10^2^ are stated as 0, Marked in red are the highest intensities for each signal, in blue the second highest

| **10^5^** | DC lamps | | RF lamps | | |
| --- | --- | --- | --- | --- | --- |
|  | Krypton | Xenon | Kr 13MHz | Kr 100kHz | Xe 13MHz |
| [H_2_O]_2_ H^+^ | 0.007 | 0 | 0.011 | 0.009 | 0 |
| [H_2_O]_3_ H^+^ | 0 | 0 | 0.002 | 0.004 | 0 |
| [Mes]^+^ | 0.258 | 0 | 0.524 | 2.818 | 0.077 |
| [Ace+H]^+^ | 0.333 | 0 | 2.459 | 0.502 | 0.137 |
| [[Ace]_2_+H]^+^ | 0.181 | 0 | 1.522 | 3.172 | 0.181 |
| [IPA+H]^+^ | 0.036 | 0 | 0.246 | 0.063 | 0.002 |
| [IPA]_2_+H]^+^ | 0.028 | 0 | 1.021 | 3.117 | 0.002 |
| [AceN+H]^+^ | 0.005 | 0 | 0.009 | 0.003 | 0 |
| [AceN+H_2_O+H]^+^ | 0.003 | 0 | 0.004 | 0.004 | 0 |
| [[AceN]_2_+H]^+^ | 0.003 | 0 | 0.014 | 0.012 | 0 |
| [[AceN+H_2_O]_2_+H]^+^ | 0 | 0 | 0 | 0 | 0 |
